# Systematic review of factors influencing patient and practitioner delay in diagnosis of upper gastrointestinal cancer

**DOI:** 10.1038/sj.bjc.6603089

**Published:** 2006-04-11

**Authors:** S Macdonald, U Macleod, N C Campbell, D Weller, E Mitchell

**Affiliations:** 1 General Practice and Primary Care, Division of Community Based Sciences, University of Glasgow, 1 Horselethill Road, Glasgow G12 9LX, UK; 2 General Practice and Primary Care, University of Aberdeen, Foresterhill Health Centre, Westburn Road, Aberdeen AB25 2AY, UK; 3 Community Health Sciences (General Practice), University of Edinburgh, 20 West Richmond Street, Edinburgh EH8 9DX, UK; 4 School of Health and Social Care, Glasgow Caledonian University, City Campus, Cowcaddens Road, Glasgow G4 0BA, UK

**Keywords:** delay, diagnosis, systematic review, upper gastrointestinal

## Abstract

As knowledge on the causation of cancers advances and new treatments are developed, early recognition and accurate diagnosis becomes increasingly important. This review focused on identifying factors influencing patient and primary care practitioner delay for upper gastrointestinal cancer. A systematic methodology was applied, including extensive searches of the literature published from 1970 to 2003, systematic data extraction, quality assessment and narrative data synthesis. Included studies were those evaluating factors associated with the time interval between a patient first noticing a cancer symptom and presenting to primary care, between a patient first presenting to primary care and being referred to secondary care, or describing an intervention designed to reduce those intervals. Twenty-five studies were included in the review. Studies reporting delay intervals demonstrated that the patient phase of delay was greater than the practitioner phase, whilst patient-related research suggests that recognition of symptom seriousness is more important than recognition of the presence of the symptom. The main factors related to practitioner delay were misdiagnosis, application and interpretation of tests, and the confounding effect of existing disease. Greater understanding of patient factors is required, along with evaluation of interventions to ensure appropriate diagnosis, examination and investigation.

Achieving a reduction in deaths from cancer is a worldwide health-care priority. As new knowledge on the causation of cancers is discovered, new treatments developed and the search for cures continues, early recognition and accurate diagnosis becomes increasingly important. This is particularly relevant in the context of primary care, where many patients present with symptoms suggestive of cancer, but where the outcome of the diagnostic process is the exclusion of cancer in the majority of cases.

Detecting and diagnosing cancer when it is at an early stage improves prognosis for many cancers (Love, 1991; Ponten *et al*, 1995; Richards *et al*, 1999; Summerton, 1999). Previous work on delay has shown that this can occur at three phases in the time from initial symptom to diagnosis. Firstly, there is the interval between first noticing a symptom and first consulting a doctor (patient delay); secondly, the interval between first consultation with a doctor and referral (practitioner delay); and finally, the time between referral and diagnosis (hospital delay) (Nichols *et al*, 1981). If a reduction in deaths from cancer is to be achieved, a greater understanding of the reasons for late and delayed diagnosis in patients with potential cancer symptoms is required. This is a significant challenge, especially for primary care, which is usually the patient's first contact with the health services and consequently, the area to which the first two phases of delay are particularly applicable.

We conducted a systematic review of the factors that influence patient and practitioner delay (Macdonald *et al*, 2004) for all adult cancers for which the UK Department of Health (DoH) has published referral guidelines, with the exception of breast cancer (Department of Health, 2000; National Institute for Health and Clinical Excellence, 2005). This paper outlines the methods used in the review and presents results for upper gastrointestinal (GI) cancers.

## MATERIALS AND METHODS

A worldwide review of the literature from 1970 to November 2003 was conducted. Extensive searches of Medline, EMBASE, CINAHL, PsycINFO, ISI Science Citation Index, ISI Social Sciences Citation Index and the International Bibliography of the Social Sciences were carried out. Supplementary searches of Proceedings First and Web of Science Proceedings were conducted to provide relevant unpublished research. The Effective Practice and Organisation of Care, Consumers and Communication and cancer-related Cochrane Collaborative Review Groups were asked for details of any potentially relevant studies. The National Research Register and the DoH Research Findings electronic Register (ReFeR) were accessed online for details of ongoing or recently completed projects, and the Medical Research Council, Cancer Research UK and the Chief Scientist Office of the Scottish Executive Health Department were contacted for details of relevant work. A database of almost 300 UK contacts with a particular interest in cancer was established, representing Scottish and English health authorities, cancer networks and cancer leads. A list of international contacts was also compiled, including similar organisations in North America, Europe, Australia and New Zealand. Requests were made to authors active in the field for studies-in-progress and unpublished work. Citations in literature reviews and articles obtained were also reviewed, as were references provided by colleagues.

### Study selection

Studies in any language and of any design were considered. Studies were selected if they focused on adult cancer and (a) the participants were individuals/groups of patients or primary care practitioners and (b) they evaluated factors associated with the time interval between a patient first noticing a cancer symptom and first presenting to a primary health-care provider, or described an intervention designed to reduce that interval or (c) they evaluated factors associated with the time interval between the patient first presenting to primary care and being referred to secondary care, or described an intervention designed to reduce that interval. We were also interested in identifying the factors associated with patient and provider behaviour, in relation to their impact on patient help-seeking or on practitioner referral, and studies that determined provider attitudes or behaviour towards referral of patients with cancer were also included, as were those that determined patient attitudes towards cancer awareness and presentation behaviour. Studies evaluating delay from presentation to treatment were not excluded until they had been reviewed to ensure that they did not differentiate between possible stages within the delay cycle. Studies that simply assessed the outcome of delay in terms of diagnosis, treatment or patient outcomes were excluded, as were those assessing costs of interventions or validity of referral decisions.

All references identified were independently assessed by two reviewers (SM, UM). Abstracts were reviewed and full texts of studies not excluded at this stage were retrieved for detailed evaluation. Discrepancies were independently validated by a third reviewer (EM). All potentially relevant studies were independently read and, if included in the review, independently rated by the same two reviewers. Where differences of opinion occurred, papers were read by the third reviewer and findings discussed until a consensus was reached.

### Strength of evidence

Methodological quality of experimental studies (randomised controlled trial, clinical trials, controlled before and after studies) was assessed for sample formation, baseline differences between groups, unit of allocation to groups, measures of outcome and follow-up (Appendix A1). Owing to the lack of controlled trials in this field, a method of scoring descriptive studies (cross-sectional, cohort, case–control, before and after studies) was also used, allowing interpretation of useful papers that would be discarded by strict adherence to Cochrane standards. Each study was scored on six criteria relevant to its design (e.g. case–control was assessed for a clear research question, source of cases/controls, clear inclusion/exclusion criteria, sampling method and comparability of the groups) (Appendix B1). Similar use of these systems has been described elsewhere (Mitchell and Sullivan, 2001).

However, many papers in this review used methodologies that did not lend themselves to the scoring systems outlined. Several included every patient attending a particular clinic and most collected data using either medical records review or structured interviews with patients. We therefore assessed each included study for the strength of its evidence in relation to the factors reported as leading to increased or decreased delay. Assessment of studies that had already been rated using the systems outlined above was based on those previous scores. Evidence was rated as ‘strong’ if a paper had an adequate sample size, used a rigorous methodology to ascertain data (i.e. not open to selection bias) and reported statistically significant differences in relation to the factors identified (or used appropriate analytic techniques if qualitative). Evidence was rated as ‘moderate’ if a paper had an adequate sample size, reported significant differences but used a less rigorous methodology to ascertain data, or had an adequate sample size, used a rigorous methodology to ascertain data but used comparative analysis or reported only relevant descriptive statistics, without performing statistical testing of differences. Finally, evidence was rated as ‘insufficient’ if a paper had an unclear or inappropriate method of ascertaining data and insufficient analysis. If a study inferred results, the strength of its evidence was reduced, for example, strong became moderate.

Narrative synthesis of findings was then carried out to identify key concepts and themes that were shared across individual studies. Textual information on cancer group, delay aspects studied and differences resulting from any intervention was also recorded.

## RESULTS

**Figure 1:**
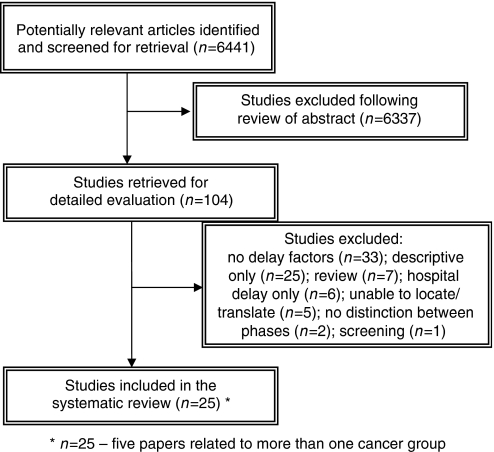
Flow of studies into the review.

The search strategy identified 6441 abstracts, which were reviewed to determine their suitability for inclusion (Figure 1). Full reprints were obtained for the 104 articles that met the inclusion criteria and these were evaluated further. Kappa co-efficient for inter-rater agreement beyond chance was 0.5. Twenty-five papers were included in the review.

The majority of included studies (*n*=18) were conducted in western Europe, almost half of these in the UK. None of the studies employed a controlled trial methodology. Most involved medical records review and/or structured interviews with patients; seven studies used mixed methods of data collection. More than half of the papers studied patient and practitioner delay factors, six studied patient factors only and six studied practitioner factors only. Prominence was given to stomach, followed by oesophagus, pancreas and small intestine. Nine papers were assessed as providing strong evidence, nine provided moderate evidence and seven provided insufficient evidence.

In general, studies were relatively small in size, involving between 30 and 2500 participants (mean 383; median 133). Only two of the studies included practitioners as participants. In the majority, the population under study was identified from secondary care (*n*=21). In seven studies participants were hospital in-patients, in four they were outpatient attenders and in two others both in-patients and outpatients were involved. A further seven studies identified patients from hospital records. One study involved members of the public, and another patients participating in an existing trial. Only three studies recruited from primary care. The period under study ranged from 1 week to 30 years.

### Delay intervals

Twenty studies reported length of delay, either from patient recognition of symptoms to first presentation (*n*=17) or from presentation to referral or diagnosis (*n*=13). Median patient delay ranged from 2 weeks to 7.5 months (Bedikan *et al*, 1979; Mikulin and Hardcastle, 1987; Zilling *et al*, 1990; Haugstvedt *et al*, 1991; Wile *et al*, 1993; Porta *et al*, 1996; Martin *et al*, 1997; Mariscal *et al*, 2001; Irving *et al*, 2002; Look *et al*, 2003) and practitioner delay from zero to just under 9 months (Mikulin and Hardcastle, 1987; Zilling *et al*, 1990; Haugstvedt *et al*, 1991; Jones and Dudgeon, 1992; Martin *et al*, 1997; Mariscal *et al*, 2001; Irving *et al*, 2002; Look *et al*, 2003).

### Factors influencing patient delay

Nineteen papers considered factors that influenced patient delay in presentation. Thirteen identified factors that increased delay and 13 identified factors that decreased delay (Table 1).

Symptoms, patient's interpretation of them and the associated impact on delay emerged as a major theme across studies. Heightened awareness of symptoms provoked more prompt presentation to a practitioner (Ojala *et al*, 1982; Delaney, 1998; Gullo *et al*, 2001), whereas lack of awareness resulted in delay (Nagao and Takahashi, 1979; Mikulin and Hardcastle, 1987; Arvanitakis *et al*, 1992; Porta *et al*, 1996; Rothwell *et al*, 1997; Ibingira, 2001). Similarly, the perceived significance of symptoms was a key factor, but the precise nature of its effect on delay was inconclusive. Many patients consulted more quickly when their symptoms were more serious or were perceived to be more serious, including the presence of pain or bleeding (Hackett *et al*, 1973; MacAdam, 1979; Ojala *et al*, 1982; Mikulin and Hardcastle, 1987; Maglinte *et al*, 1991; Grannell *et al*, 2001; Mariscal *et al*, 2001). For others, however, experiencing pain increased their delay (Hackett *et al*, 1973; Ibingira, 2001; Look *et al*, 2003), as did weight loss (Haugstvedt *et al*, 1991). The presence of multiple symptoms or comorbidity resulted in more prompt presentation (Porta *et al*, 1996; Mariscal *et al*, 2001).

The fear associated with recognising potential cancer symptoms was found to have both a positive (Delaney, 1998) and negative (Hackett *et al*, 1973) impact on presentation behaviour. Although the associated worry could be beneficial in reducing delay, we also found evidence that patients were less likely to consult when they were afraid that their symptoms were indicative of cancer (Mikulin and Hardcastle, 1987) or meant that they would have to undergo tests (Delaney, 1998). Patients who redefined their symptoms, perhaps in response to that fear, delayed longer (Delaney, 1998).

There was some evidence to suggest that the setting of first presentation impacts on delay, with those who presented first to hospital doing so after shorter symptom duration (Wile *et al*, 1993; Mariscal *et al*, 2001) (Table 2).

A number of studies considered the relationship between certain patient characteristics and presentation behaviour. Ethnicity was associated with reduced delay (Wile *et al*, 1993), while lower socio-economic status (Hackett *et al*, 1973; Mikulin and Hardcastle, 1987; Porta *et al*, 1996) was associated with increased delay. By and large, sex had no impact on delay (MacAdam, 1979; Mikulin and Hardcastle, 1987; Porta *et al*, 1996; Mariscal *et al*, 2001), whereas the evidence presented for education level (Porta *et al*, 1996; Mariscal *et al*, 2001) and age was inconclusive (Bedikan *et al*, 1979; MacAdam, 1979; Mikulin and Hardcastle, 1987; Zilling *et al*, 1990; Porta *et al*, 1996; Mariscal *et al*, 2001; Look *et al*, 2003) (Table 2).

### Factors influencing practitioner delay

Nineteen studies considered factors that influenced practitioner delay in referral. Seventeen identified factors that increased delay and six identified factors that decreased delay (Table 1).

Delay in referral was primarily related to initial misdiagnosis of a common symptom or failure to make a diagnosis at the initial encounter with the patient (Bedikan *et al*, 1979; Nagao and Takahashi, 1979; Ojala *et al*, 1982; Hallissey *et al*, 1990; Arvanitakis *et al*, 1992; Rothwell *et al*, 1997). Moreover, referral was likely to be delayed if the patient was being treated for a benign condition, particularly with acid suppression (Mikulin and Hardcastle, 1987; Rothwell *et al*, 1997; Bramble *et al*, 2000). Inappropriate application of tests, inaccurate test results and previous receipt of negative test results were additional causes of delay (Zilling *et al*, 1990; Maglinte *et al*, 1991; Wile *et al*, 1993; Rothwell *et al*, 1997; Look *et al*, 2003) (Table 3).

Some of the more recent studies evaluated policy initiatives aimed at tackling referral delays. Where a rapid access endoscopy service was available, patients were found to experience less delay in referral (Martin *et al*, 1997; Manes *et al*, 2002), albeit with the caveat that inappropriate use of such a service could actually contribute to delay (Manes *et al*, 2002). Similarly, the introduction of the DoH cancer referral guidelines was found to reduce delay (Irving *et al*, 2002).

Evaluation of the impact of tumour site on delay demonstrated that patients with oesophageal cancer were more likely to experience delayed referral than patients with stomach cancer (Martin *et al*, 1997). In addition, patients with any upper GI cancer, regardless of site, were delayed more than patients with colorectal or other cancers (MacAdam, 1979; Jones and Dudgeon, 1992).

Only a limited number of patient characteristics were considered in relation to practitioner delay. Male patients (Zilling *et al*, 1990; Haugstvedt *et al*, 1991), older patients (Mikulin and Hardcastle, 1987) and patients from lower socio-economic groups (Mikulin and Hardcastle, 1987) were less likely to experience delayed referral (Table 3).

## DISCUSSION

In 1938, the American Journal of Cancer published a paper by Pack and Gallo (1938) entitled ‘The culpability for delay in the treatment of cancer’. This was one of the first papers to study delay as it has been considered in this review. Sadly, almost 70 years later, we echo their findings. Analysis of the studies included in this review shows that there are associations between at least 14 different factors and delay. Five of these concerned delay by patients; nine concerned delay by practitioners. The main themes to emerge related to recognition and interpretation of symptoms, patient history, patient characteristics and health-care factors.

### Quality of studies

This was an extensive and comprehensive review of the world literature; yet, although we identified an abundance of descriptive work, few evaluative studies have been carried out. Great variation in study design and quality precluded a meta-analysis; rather, we graded study evidence by the robustness of its methodology and analysis, allowing us to weight each study appropriately in our overall assessment of delay-related factors. Any critique of this type can only be based on the information that has been reported and in many cases this was incomplete and unclear. However, more recent studies tended to rate more highly, possibly a reflection of the increased reporting standards now required by journals. Although the majority of studies used a non-traditional design and conducted records review or structured patient interview, they were on the whole methodologically rigorous. Nine of the 25 studies provided strong evidence and a further nine moderate evidence. Only seven studies were graded as insufficient.

### Delay intervals

The majority of included studies reported length of delay, and although more than half obtained these data from abstraction of hospital records, there was no uniform approach to the provision of this information. Consequently, it was not possible to determine definitive delay intervals. Nonetheless, the available data do demonstrate extreme delay in both the patient and practitioner phases. What is also clear is that delay intervals are not decreasing; those recorded in the last 5 years are as lengthy as those recorded 20 years ago. Despite this, we identified no intervention studies related to upper GI cancers.

### Symptom recognition

The results of this review suggest that what is important in terms of patient delay is recognition of the seriousness of a symptom, and not simply recognition of the presence of the symptom itself. Patients who have significant symptoms, and perceive them to be so, may present promptly. Equally, they may delay through fear. The catalyst for presentation was often when a symptom became debilitating or hampered normal activities, but the precise influence of this was unclear. Experiencing pain, for example, was found to both reduce and increase patient delay. An obvious difficulty, particularly with GI cancers, is that common symptoms can be attributable to benign disease. Consequently, patients may not present immediately. This poses a challenge for health educators, who must strike a balance between emphasising the potentially significant nature of symptoms, regardless of their commonality, and creating unnecessary fear.

Similarly, symptom recognition and interpretation were no less important for practitioners. Delays following initial patient presentation were often the result of misdiagnosis of benign disease or of adopting a wait and see approach following the first encounter with the patient.

### Patient history

As could perhaps be predicted, patients’ previous experience played a role in current decisions to consult with cancer symptoms. Perceptions of symptom seriousness were often based on a personal or family history of similar symptoms and this could have a two-fold effect. Such knowledge prompted some patients to present sooner rather than later; however, for some, the fear that symptoms were indicative of cancer resulted in delayed consultation. Patients who redefined their symptoms, perhaps in relation to that fear or on the basis of prior experience, delayed longer. Similarly, patients who had previously been treated for benign disease delayed longer. Conversely, patients with comorbidity presented more quickly, possibly as a consequence of regular attendance for other conditions, and the resultant ease with which new symptoms could be discussed.

In addition, patient history was a significant factor in relation to practitioner behaviour. Although research relating to the impact of coexisting disease or frequent patient attendance was inconclusive, referral was more likely to be delayed if the patient was being treated for a benign condition. Delays were also demonstrated where patients had already been investigated for the same or similar symptoms and had previous negative test results. Whilst it is undoubtedly appropriate for practitioners to consider past history when considering new symptoms or diagnoses, this does raise the question of when a negative test result should be considered to be no longer of relevance.

### Patient characteristics

The potential for individual patient characteristics to impact on delay was considered, to some extent, in the included studies, and analysis of these suggests that lower socio-economic status increases delay, whereas sex has no impact. However, there remains a dearth of evidence relating to why some patients respond to early warning signs or unusual symptoms, whilst, others do not. Accordingly, there is a prevailing need to understand the impact of social and psychological factors on patient behaviour. Many studies focused on the appropriateness of patient response to symptoms, yet only a minority attempted to explore the reasons for this, and then primarily from a psychological perspective. Few have done so from a social perspective. As a consequence, there is little conclusive evidence relating to the influence brought to bear by age, level of education or social network. Yet if we consider that new symptoms occur, not in isolation, but in the context of an individual's life circumstances, it is likely that investigations of social factors will yield valuable insights in this area.

Although only a limited number of patient characteristics were considered in relation to practitioner referral behaviour, there was some evidence to suggest that younger and female patients experienced increased delays. Conversely, patients from lower socio-economic groups were less likely to experience delay. This is in direct contrast to the impact of socio-economic status on patient delay, and it could be postulated that as these patients have already delayed prior to their initial presentation, the need for rapid referral is all the more apparent.

### Health-care factors

Several recent policy initiatives have been directed at the practitioner phase of delay, and the results of this review suggests that these may be starting to have an impact. An evaluation of outcomes related to the introduction of standardised referral guidelines in the UK demonstrated positive effects on delay. Similarly, a relationship between provision of services and reduced delay has also been shown, with those patients who have use of a rapid access endoscopy service experiencing less delay in referral. However, the evidence in this regard is as yet inconclusive, and it had been suggested that inappropriate use of such a service could increase delay as a consequence of an increased volume of referrals. As yet, only a few studies have explored these initiatives in detail, and although those that have done so indicate that they are, for the most part, successful, further work will enable more definitive conclusions to be drawn.

### Implications for practitioners

Many of the studies in this review reported practitioner misdiagnosis as a significant contributor to delay. The studies were not designed in a way that would allow the observer to assess the nature of that ‘misdiagnosis’. However, it is clear that there are significant challenges for practitioners in assessing symptoms such as dyspepsia, including evaluation in the context of acid suppression therapy, and previous negative test results. Such issues need to be clearly addressed within guidelines.

### Implications for research

One of the most surprising findings of this review is that although the majority of studies reported on primary care practitioner delay, only two of these involved primary care practitioners as participants and only three recruited patients from primary care. Most were conducted in secondary care and consisted of a series of either prospective or retrospective clinic experiences. Furthermore, in those studies involving practitioners, the participants were primary care doctors. Consequently, the contribution that may be made by other health-care practitioners, such as nurses, has not yet been investigated.

Ramirez *et al* (1999), in their review of factors predicting delayed presentation with breast cancer, highlighted the need for further research into understanding delay in order to impact on presentation behaviour. Although some work has been commissioned since their call for it, it is clear that we still lack understanding of this area. This review demonstrates the complex nature of delay and consequently, the difficulty of devising strategies to reduce it. However, this must be achieved if the ultimate aim of improving survival is to be met. Influencing this is likely to involve gaining greater appreciation of the impact of patient characteristics, alongside development of strategies to aid practitioners in assessing GI symptoms.

## Figures and Tables

**Table 1:** Main delay factors and assessment of evidence

	**No. of studies (subjects)**	**Supported (studies)**	**Not supported (studies)**	**No impact (studies)**	**Overall assessment /conclusion**
*Increases patient delay*					
Non-recognition of symptom seriousness	9 (1840)	2S, 3M, 4I	—	—	Increases delay
Cancer site – stomach	2 (713)	1S, 1M	—	—	Increases delay
Lower socio-economic status	4 (979)	2S, 1M	—	1M	Increases delay
Comorbidity	2 (400)	—	2S	—	Reduces delay
First presenting to hospital	2 (266)	—	2S	—	Reduces delay
Male sex	5 (797)	1I	1S	1S, 2M	No impact on delay
Fear	3 (1271)^a^	2S, 1M	2S	—	Inconclusive
Experiencing pain	7 (1169)	1S, 1M, 1I	1S, 3M	—	Inconclusive
Older age	7 (800)	1S, 1M	1S, 2M	1S, 1M	Inconclusive
Lower education	2 (400)	1S	1S	—	Inconclusive
Family history	3 (777)	1S	1S	1S	Inconclusive
					
*Increases practitioner delay*					
Initial misdiagnosis	6 (3556)	2M, 4I	—	—	Increases delay
Acid suppression treatment	3 (316)	1S, 2M	—	—	Increases delay
Inappropriate/inaccurate tests	3 (226)	1S, 2M	—	—	Increases delay
Previous negative test result	2 (94)	1S, 1M	—	—	Increases delay
Cancer site – oesophagus	2 (1580)	1M, 1I	—	—	Increases delay
Female patient	2 (1215)	1S, 1M	—	—	Increases delay
Older patient age	1 (83)	—	1M	—	Reduces delay
Lower patient socio-economic status	1 (83)	—	1M	—	Reduces delay
Use of referral guidelines	1 (90)	—	1S	—	Reduces delay
Frequent patient attendance	2 (265)	1I	—	1M	Inconclusive
Comorbidity	2 (267)	1S	1S	—	Inconclusive
Use of rapid access endoscopy	2 (821)	1S	1I	—	Inconclusive

a Paper reports conflicting evidence (i.e. which both supports and refutes the factor as a cause of delay).

S=strong evidence; M=moderate evidence; I=insufficient evidence (based on the methodological adequacy of the study).

**Table 2:** Patient-associated delay factors

**Author(s)**	**Location**	**Study type**	**Participants**	**Cancer site**	**Factors that increase delay**	**Factors that decrease delay**	**No impact on delay**	**Strength of evidence**
Hackett *et al* (1973)	Massachusetts, USA	Prospective observational	563 patients (aged 17–91; mean 62; 46% men, 54% women), 6% with stomach cancer	Stomach	Symptom type – pain; cancer site – stomach; social class – lower; worry over health; family history	Worry; incapacitated by symptoms; acknowledgment of cancer		Strong
Bedikan *et al* (1979)	Texas, USA	Retrospective observational	73 patients (aged <40; 48% men, 52% women)	Stomach		Age – older		Moderate
Nagao and Takahashi (1979)	Japan	Retrospective observational	536 patients	Stomach	Non-recognition of symptom seriousness^a^			Insufficient
MacAdam (1979)	England	Prospective observational	150 patients (21% with stomach cancer), 105 GPs	Stomach	Cancer site – stomach	Symptom type – abdominal pain, bleeding	Socio-economic status; age; sex; social isolation; frequency of consulting	Moderate
Ojala *et al* (1982)	Finland	Retrospective observational	162 patients (aged 38–82, mean 63; 59% men, 41% women)	Oesophagus		Patient awareness; symptom type – dysphagia^a^		Insufficient
Mikulin and Hardcastle (1987)	England	Prospective observational	83 patients (mean 71; 64% men, 36% women)	Stomach	Non-recognition of symptom seriousness; symptom type – no pain; fear; age – older; social class – lower	Age – younger	Sex	Moderate
Zilling *et al* (1990)	Sweden	Prospective observational	50 patients (aged 31–85, mean 68; 74% men, 26% women)	Stomach	Age – younger			Strong
Haugstvedt *et al* (1991)	Norway	Prospective observational	1165 patients	Stomach	Symptom type – weight loss	Referral to university hospital		Moderate
Maglinte *et al* (1991)	Indiana, USA	Retrospective observational	77 patients (aged 30–89, mean 59; 64% men, 36% women	Small intestine		Symptom type – pain, bleeding		Moderate
Arvanitakis *et al* (1992)	Greece	Observational	100 patients (aged 40–90; 64% men, 36% women)	Stomach	Non-recognition of symptom seriousness			Insufficient
Wile *et al* (1993)	California, USA	Retrospective observational	49 patients (median 57; 45% men, 55% women)	Stomach		First presenting at hospital; ethnicity – minority groups		Strong
Porta *et al* (1996)	Spain	Prospective observational	183 patients (mean 67; 66% men, 34% women)	Oesophagus, stomach, duodenum	Age – older; sex – male; illiteracy; social class – lower; unemployment; non-recognition of symptom seriousness	Age – younger; comorbidity; recognition of symptom seriousness	Marital status; family history	Strong
Rothwell *et al* (1997)	Ireland	Prospective observational	100 patients (aged 37–83, median 69; 70% men, 30% women)	Oesophagus	Non-recognition of symptom seriousness			Moderate
Delaney (1998)	England	Qualitative interviews	31 patients with dyspepsia (aged 50+, mean 64; 52% men, 48% women)	Stomach	Fear of investigation; symptom re-definition; fatalism	Recognition of symptom seriousness; personal or family history; fear of cancer		Strong
Mariscal *et al* (2001)	Spain	Prospective observational	217 patients (aged 59–74, mean 65; 59% men, 41% women), 27% with upper GI cancer	Oesophagus, stomach	Education level – higher	Comorbidity; symptom type – pain, bleeding; first presenting at hospital; multiple symptoms	Age; sex; availability of vehicle	Strong
Gullo *et al* (2001)	Italy	Case–control	305 patients (aged 30–75, mean 61; 62% men, 38% women) and 305 matched controls	Pancreas		Symptom recognition^a^		Moderate
Ibingira (2001)	Uganda	Prospective observational	35 patients (aged 34–78; 77% men, 23% women)	Stomach	Non-recognition of symptom seriousness; symptom type – pain; acid suppression treatment			Insufficient
Grannell *et al* (2001)	Ireland	Cross-sectional	164 members of the public (93 aged <45, 71 aged 45+; 51% men, 49% women)	Oesophagus	Sex – female	Increased awareness – dysphagia^a^		Insufficient
Look *et al* (2003)	Singapore	Retrospective observational	44 patients (aged 36–83, mean 67; 70% men, 30% women)	Stomach	Age – younger; symptom type – pain			Moderate

Abbreviation: GI=gastrointestinal.

a Study infers findings.

**Table 3:** Practitioner-associated delay factors

**Author(s)**	**Location**	**Study type**	**Participants**	**Cancer site**	**Factors that increase delay**	**Factors that decrease delay**	**No impact on delay**	**Strength of evidence**
Bedikan *et al* (1979)	Texas, USA	Retrospective observational	73 patients (aged <40; 48% men, 52% women)	Stomach	Initial misdiagnosis			Moderate
Nagao and Takahashi (1979)	Japan	Retrospective observational	536 patients	Stomach	Initial misdiagnosis			Insufficient
MacAdam (1979)	England	Prospective observational	150 patients (21% with stomach cancer), 105 GPs	Stomach		Cancer site – stomach	Regular consulting rate of patient	Moderate
Ojala *et al* (1982)	Finland	Retrospective observational	162 patients (aged 38–82, mean 63; 59% men, 41% women)	Oesophagus	Initial misdiagnosis			Insufficient
Mikulin and Hardcastle (1987)	England	Prospective observational	83 patients (mean 71; 64% men, 36% women)	Stomach	Acid suppression treatment; patient age – younger	Patient age – older; patient social class – lower		Moderate
Zilling *et al* (1990)	Sweden	Prospective observational	50 patients (aged 31–85, mean 68; 74% men, 26% women)	Stomach	Patient sex – female; comorbidity; previously negative results	Patient sex – male		Strong
Hallissey *et al* (1990)	England	Cohort	2585 patients with dyspepsia (aged 40+)	Stomach	Initial misdiagnosis			Insufficient
Haugstvedt *et al* (1991)	Norway	Prospective observational	1165 patients	Stomach	Patient sex – female	Referral to university hospital		Moderate
Maglinte *et al* (1991)	Indiana, USA	Prospective observational	77 patients (aged 30–89, mean 59; 64% men, 36% women	Small intestine	Inappropriate tests			Moderate
Jones and Dudgeon (1992)	England	Retrospective observational	245 GPs, 1465 patients (>60 with upper GI cancer)	Oesophagus, stomach	Cancer site – oesophagus			Moderate
Arvanitakis *et al* (1992)	Greece	Observational	100 patients (aged 40–90; 64% men, 36% women)	Stomach	Initial misdiagnosis			Insufficient
Wile *et al* (1993)	California, USA	Retrospective observational	49 patients (median 57; 45% men, 55% women)	Stomach	Inaccurate tests			Strong
Martin *et al* (1997)	England	Prospective observational	115 patients (aged 31–89, median 66; 61% men, 39% women)	Oesophagus, stomach	Frequent attendance by patient; cancer site – oesophagus	Access to rapid screening (open access endoscopy)	Initial symptom	Insufficient
Rothwell *et al* (1997)	Ireland	Prospective observational	100 patients (aged 37–83, median 69; 70% men, 30% women)	Oesophagus	Acid suppression treatment; initial misdiagnosis; inappropriate tests			Moderate
Bramble *et al* (2000)	England	Retrospective observational	133 patients (aged 38–97, mean 69; 53% men, 47% women)	Oesophagus, stomach	Acid suppression treatment			Strong
Mariscal *et al* (2001)	Spain	Prospective observational	217 patients (aged 59–74, mean 65; 59% men, 41% women), 27% with upper GI cancer	Oesophagus, stomach		Comorbidity; symptom type – pain, bleeding		Strong
Irving *et al* (2002)	England	Observational	90 patients (72% with oesophageal, 28% with gastric)	Oesophagus, stomach		Use of referral guidelines; 2-week rule		Strong
Manes *et al* (2002)	Italy	Cross-sectional	706 endoscopy referrals (aged 15–86, mean 47; 55% men, 45% women)	Stomach	Inappropriate use of endoscopy^a^			Strong
Look *et al* (2003)	Singapore	Retrospective observational	44 patients (aged 36–83, mean 67; 70% men, 30% women)	Stomach	Previously negative results			Moderate

Abbreviation: GI=gastrointestinal.

a Study infers findings.

**Table A1:** 

	**Notes**
*Sample formation*	
2 – Random	• For example, stratification
1 – Quasi random	• Randomised by toss of coin – 1
	• Group members acting as both intervention and control (if randomised) – 1
0 – Selected, concurrent or historical	• Subjects chosen
	
*Baseline differences*	
2 – None or adjusted	
1 – Differences unadjusted	Mentioned but not specified (e.g. no difference) – 1
0 – No statement	
*N.B*. This is the difference between groups providing the unit of analysis	
	
*Unit of allocation*	
2 – Practice/clinic	
1 – Doctor	Nothing of note
0 – Patient	
	
*Outcome measures*	
2 – Objective/subjective with assessors blinded	
1 – Subjective, assessors not blinded, explicit criteria given	• Questionnaires – 1
	• Mentions ‘eligible’ patients but does not specify what eligible is (open to interpretation) - 1 (or based on scores etc.)
	• Mentions that outcomes may be under/over estimated
0 – Subjective, assessors not blinded, no explicit criteria	
	
*Follow-up*	
2 – >90% subjects starting study	• Is information provided to verify that researchers know that all subjects were contacted
1 – 80–90% subjects starting study	Exclude from follow-up calculation:
	• Subjects excluded from analysis
0 – <80% subjects starting study	• Non-responders in questionnaire surveys
	• If there is no statement about follow-up or conclusive information in tables (e.g. baseline n =..; follow-up n=…) do not calculate. Follow-up = unable to determine
*N.B*. This relates to subjects accounted for at the end of the study, not just those with a positive outcome.	

**Table B1:** 

**Source of case**	**Duration/intensity of exposure**	**Validity of measures**	**Quality control**	**Subject ‘compliance’ rate**	**Postintervention data**
*Case report*					
1 – Identified	1 – Information	1 – Statement	1 – Statement	1 – Information	1 – Given
0 – Not identified	0 – No information	0 – No statement	0 – No statement	0 – No information	0 – Not given
					
**Source of cases**	**Inclusion/exclusion criteria**	**Sampling method**	**Duration/intensity of exposure**	**Validity of measures**	**Subjects lost to follow-up**
*Case series*					
1 – Identified	1 – Statement	1 – Given	1 – Information	1 – Statement	1 – Information
0 – Not identified	0 – No statement	0 – Not given	0 – No information	0 – No statement	0 – No information
					
**Research Q or hypothesis**	**Source of cases**	**Inclusion/exclusion criteria**	**Sample size**	**Dealing with bias or confounders**	**Analytic methods**
*Cross-sectional study*					
1 – Statement	1 – Identified	1 – Statement	1 – Statement	1 – Information	1 – Description
0 – No statement	0 – Not identified	0 – No statement	0 – No statement	0 – No information	0 – No description
					
**Research Q or hypothesis**	**Source of cases**	**Inclusion/exclusion criteria**	**Nonresponse rate**	**Starting point for each subject**	**Duration/intensity of exposure**
*Cohort study*					
1 – Statement	1 – Identified	1 – Statement	1 – Statement	1 – Definition	1 – Information
0 – No statement	0 – Not identified	0 – No statement	0 – No statement	0 – No definition	0 – No information
					
**Research Q or hypothesis**	**Source of cases**	**Source of controls**	**Inclusion/exclusion criteria**	**Sampling method**	**Comparability of control group**
*Case–control study*					
1 – Statement	1 – Identified	1 – Identified	1 – Statement	1 – Given	1 – Statement
0 – No statement	0 – Not identified	0 – Not identified	0 – No statement	0 – Not given	0 – No statement
					
**Research Q or hypothesis**	**Source of cases**	**Inclusion/exclusion criteria**	**Sample size**	**Starting point for each subject**	**Validity of measures**
*Before and after study*					
1 – Statement	1 – Identified	1 – Statement	1 – Statement	1 – Definition	1 – Statement
0 – No statement	0 – Not identified	0 – No statement	0 – No statement	0 – No definition	0 – No statement
• If simply states‘ to evaluate’ = 0				• For patients - disease	• Explicit statement, or face validity
				• For GPs starting point; background; where evaluation is coming from	(comparison to ‘gold standard’ would be ideal, but we accepted less in these designs)

## Appendix A1

### Scoring system – experimental studies

(Randomised controlled trials/case-controlled trials/controlled before and after studies) Table A1

## Appendix B1

### Scoring system – descriptive studies

Table B1
